# Sublingual pythiosis in a cat

**DOI:** 10.1186/s13028-017-0330-z

**Published:** 2017-09-26

**Authors:** Jessica Sonia Fortin, Michael John Calcutt, Dae Young Kim

**Affiliations:** 1 Veterinary Medical Diagnostic Laboratory, University of Missouri, 810 E Campus Loop, Columbia, MO USA; 2 Department of Veterinary Pathobiology, University of Missouri, 313 Connaway Hall, Columbia, MO USA

**Keywords:** Eosinophilic granulomatous inflammation, Feline, Hyphae, *Pythium insidiosum*, Sublingual mass

## Abstract

**Background:**

Pythiosis is a potentially fatal but non-contagious disease affecting humans and animals living in tropical and subtropical climates, but is also reasonably widespread in temperate climates, throughout the world. The most commonly reported affected animal species with pythiosis are equine and canine, with fewer cases in bovine and feline. Extracutaneous infections caused by *Pythium insidiosum* have been rarely described in the cat.

**Case presentation:**

Sublingual pythiosis was diagnosed in a 2-year-old, male, Domestic Shorthair cat. The cat had a multilobulated, sublingual mass present for 3 months. Histopathological examination revealed severe multifocal coalescing eosinophilic granulomatous inflammation. Centers of the inflammation contained hyphae that were 3–7 μm-wide, non-parallel, uncommonly septate and rarely branching. The fungal-like organism was identified as *P. insidiosum* by polymerase chain reaction and subsequent amplicon sequencing.

**Conclusions:**

Only a few feline pythiosis cases have been reported and, when encountered, it usually causes granulomatous diseases of the skin or gastrointestinal tract. This case presents an unusual manifestation of feline pythiosis, representing the first involving the oral cavity in cats or dogs.

## Background

Pythiosis is a potentially fatal but non-contagious disease affecting humans and animals living in tropical and subtropical climates, but that is emerging and now reasonably widespread in temperate climates, throughout the world. Notably, it has been reported in other countries such as the Republic of Korea, Japan and Haiti [1]. The infection is contracted typically during the warm months, from early spring to late fall [2]. Pythiosis, caused by *Pythium insidiosum*, represents a life-threatening infection in humans and mammals. Members of the *Pythium* genus, class Oomycota, are water or soil dwelling and phylogenetically distinct from fungi, rather closely related to diatoms and algae [3]. Many *Pythium* sp. are economically important plant pathogens [1, 4]. *Pythium insidiosum* is the only mammalian pathogen implicated previously in this genus. Over the last decade, a case of *Pythium aphanidermatum* invasive wound infection has been reported in an injured man following combat trauma in Afghanistan [5]. Animals affected by the disease are often younger and exposed to warm, freshwater habitats [1]. The infective form is the asexual motile zoospore produced in aquatic environs hypothetically in association with plant materials that presumably causes infection by encysting on injured skin or a mucosal surface [1, 2, 4, 6]. Definitive identification relies on serology, immunohistochemistry and molecular diagnostics, but not solely on morphological features, since histopathological features of fungal pathogens such as coenocytic fungi are similar [7, 8].

Pythiosis was first identified in the horse during the 19th century [4]. The first reported case of pythiosis in a dog, cat and human occurred respectively in 1971 [9], 1991 [10] and 1989 [11]. In animals, the infection exhibits different clinical presentation in cutaneous, subcutaneous, intestinal, and disseminated manifestations depending on the route of entry. Skin infection is most common in animals. Interestingly, in dogs, gastrointestinal pythiosis occurs more frequently than the cutaneous/subcutaneous clinical presentation [6]. Pythiosis occurs most frequently in horses and dogs, and only a “handful” of feline cases have been reported. The purpose of this case report is to describe an unusual manifestation of feline pythiosis, representing the first involving the oral cavity in pet animals. The general aim is to promote global monitoring of potentially emerging fungal-like diseases in uncommonly affected animal species since moving and travelling with pets is common presently.

## Case presentation

**Fig. 1:**
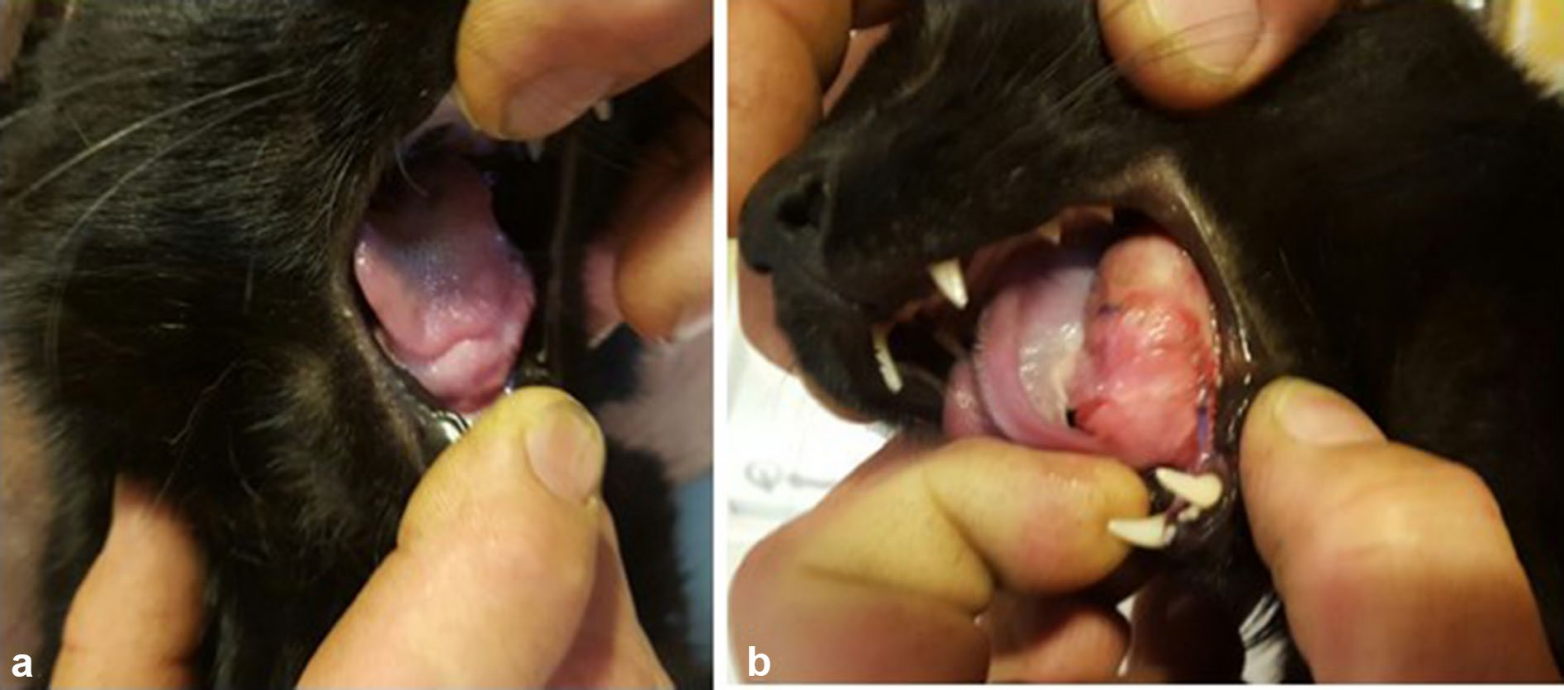
Lateral views of the sublingual mass 5 days after the biopsy (**a** right, **b** left). A 2-year-old, male, Domestic Shorthair cat had a multilobulated, sublingual mass present for 3 months. Sublingual pythiosis was diagnosed following histopathological examination from a biopsy specimen

A 2-year-old, male, Domestic Shorthair cat was presented at a central Missouri veterinary clinic with a multilobular, irregular and firm sublingual mass measuring 2.5 × 2 × 1 cm (Fig. 1). The cat was an indoor/outdoor cat and had no travel history. The mass was present for 3 months of duration, according to the owner, prior to consultation. No pharmacological treatment was attempted. Incisional biopsy was performed to obtain a diagnosis. The formalin-fixed specimen from the mass was submitted for histopathologic evaluation to the University of Missouri Veterinary Medical Diagnostic Laboratory, USA.

**Fig. 2:**
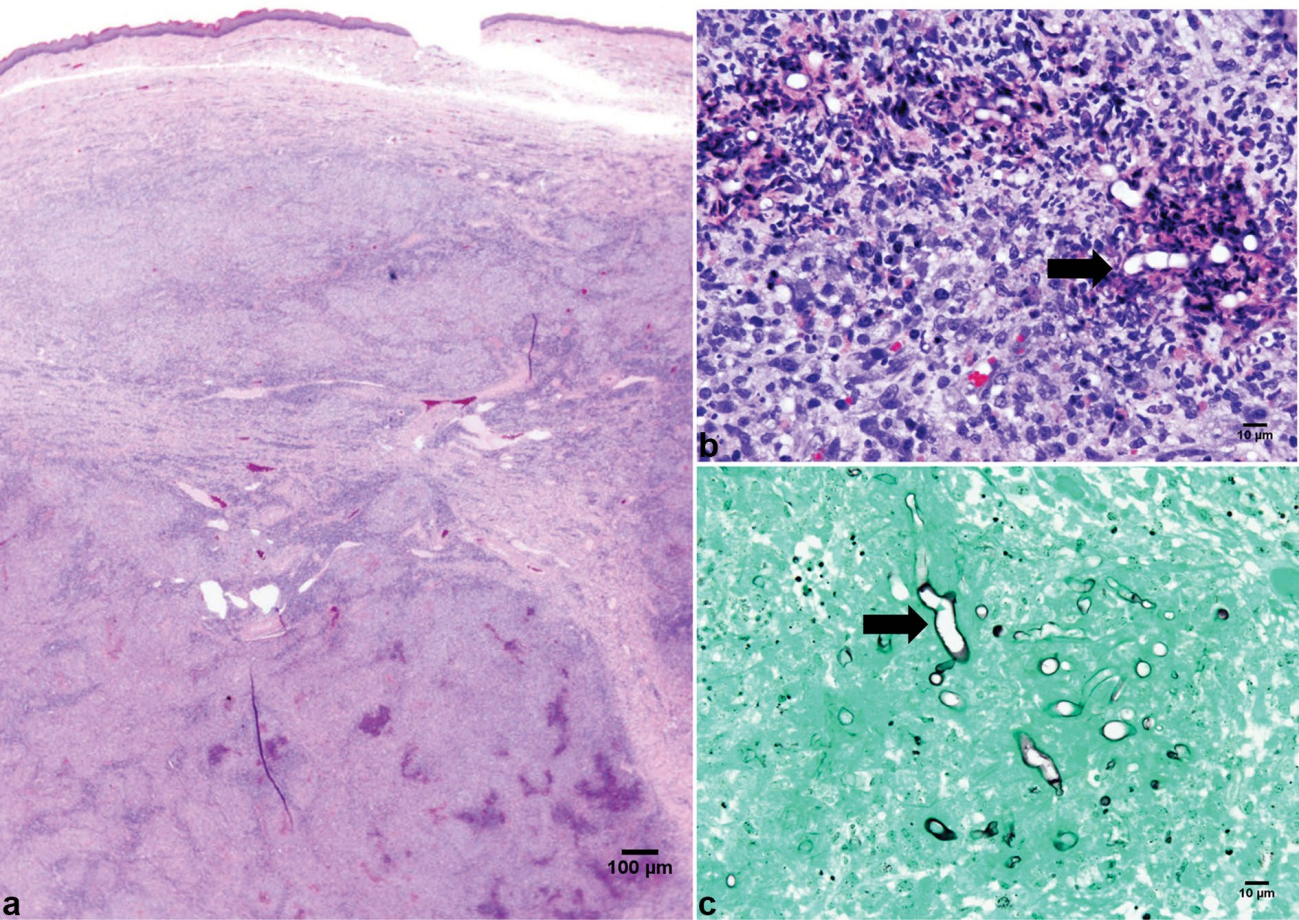
Photomicrographs of the intralesional fungal hyphae (black arrow) within the severe sublingual eosinophilic granulomatous inflammation. **a** Lower magnification of the sublingual mass composed of eosinophilic granulomatous inflammation. **b** The hyphae were 3–7 μm-wide, non-parallel, occasionally septate with rare branching. Hematoxylin and eosin (HE) stain. **c** Hyphal structures are evidentiated with Gomori methenamine silver (GMS) stain

The mass was fixed in 10% neutral buffered formalin, trimmed and embedded in paraffin. Paraffin sections (4 µm) were stained with hematoxylin and eosin (HE). Histopathological examination revealed severe multifocal to coalescing areas of eosinophilic granulomatous inflammation, characterized by multifocal central areas of necrosis with degenerated eosinophils, which were surrounded by large numbers of macrophages, epithelioid macrophages, lymphocytes and plasma cells and occasional multinucleated cells (Fig. 2a, b). The inflammation extended to the underlying skeletal muscle. The central areas of necrosis contained faintly stained fungal-like hyphae. The hyphae were 3–7 μm-wide, non-parallel, uncommonly septate and rarely branching. Hyphal structures were partially evidentiated with a Gomori methenamine silver (GMS) stain (Fig. 2c). The hyphae are morphologically similar to those of oomycetes. Based on the microscopic characteristics of the inflammation and intralesional hyphal structures, *P. insidiosum* infection was highly suspected. Although not encountered in the cat and infection of the oral cavity has not been reported, lagenidiosis was also considered as a differential diagnosis because of close similarities of microscopic lesions and hyphae to pythiosis [1]. The inflammation containing hyphal organisms reached the surgical margins so that it was unclear whether the lesion was completely excised or not. The histopathologic evaluation was summarized as a severe eosinophilic granulomatous inflammation with intralesional fungal hyphae.

The identification of the fungal-like organism as *P. insidiosum* was carried out by polymerase chain reaction (PCR) and amplicon sequencing using DNA extracted from formalin-fixed paraffin-embedded tissue [12]. PCR was carried out using the Expand Long Template PCR System (Roche, Indianapolis, IN, USA) with oligonucleotide primers that amplify the complete ribosomal Internal Transcribed Spacer 1 (ITS1) region. The resulting amplicon was purified by spin chromatography (QIAquick PCR Purification Kit, Qiagen, Valencia, CA, USA) and nucleotide sequence determined using BigDye terminator chemistry with the amplification primers at the University of Missouri DNA Core Facility. Sequence analysis disclosed the identity of the pathogen as *P. insidiosum*. The sequence of the complete ITS1 region amplified from the cat tissue (GenBank accession MF928597) was > 99% identical to multiple GenBank entries from *P. insidiosum* (identified by using Blastn). ITS1 sequences from three strains submitted to GenBank as *Pythium destruens* exhibited 93–94% identity to that described herein and those from *Pythium grandisporangium* were < 82% identical to the sequence obtained from the cat lesion.

## Discussion

Only a few feline pythiosis cases have been reported wherein cutaneous, subcutaneous, retrobulbar and/or nasopharyngeal, and gastrointestinal diseases in domestic cats are described [1, 4, 10, 13]. A gastrointestinal infection was documented in two cats [13]. The oomycote *P. insidiosum* has also been described as a rare cause of gastrointestinal [Bengal tiger (*Panthera tigris*)] and pulmonary [Central American jaguar (*Panthera onca*)] diseases in nondomestic Felidae [1, 4]. There is one record of animals infected with an oral form of pythiosis. A case of pythiosis involving the hard palate and nasal cavity in a sheep from southwestern Paraná, southern Brazil, was confirmed by indirect enzyme-linked immunosorbent assay and immunohistochemistry [14]. Interestingly, two cases of canine chronic esophagitis caused by *P. insidiosum* occurred previously in rural Tennessee, USA [15].

Cases of oral cavity pythiosis have not been diagnosed in cats, here-to-fore. The clinical diagnosis of this case demonstrates that, in cat, pythiosis may affect the oral cavity, causing severe gross and microscopic lesions similar to those reported in other species involving cutaneous or subcutaneous pythiosis. Herein, the infection was limited to the oral cavity without associated skin or systemic involvement. However, for most cases of gastrointestinal/alimentary pythiosis, the prognosis is guarded or poor.

The most commonly observed lesions are attributed to direct contact with water. The present cat was an indoor/outdoor cat, which is common in rural Missouri environment, and had no history of traveling. Although the owner was skeptical, it was uncertain whether the cat had a direct contact with pond water or not. It is probable that in the oral form of the disease, the infection occurs by direct contact of the oral mucosa while drinking water contaminated by motile zoospores of *P. insidiosum* [16, 17]. It is not clear whether the involvement of the sublingual site had resulted from a foreign body such as a puncture object.

The diagnosis of pythiosis is based on morphological features of the organism on cytologic and/or histopathologic examination. The diagnosis of pythiosis cannot be made on the basis of morphologic features alone but require additional diagnostics. Fungal hyphae that are demonstrated in the granulomas with infiltrates of eosinophils should raise the suspicion for pythiosis. However, definitive identification relies on culture, molecular tools or immuno-histochemistry. Culture identification of this oomycote is time consuming and requires expertise to adequately identify the agent and avoid misdiagnosis with *Aspergillus* spp. or zygomycetes. Serology may be useful to detect antibody against *P. insidiosum* but false positive (if previously exposed) or false negative (in case of low immunogenicity such as in ocular infection) can occur [18]. Molecular techniques have been used successfully and with increasing frequency for diagnosis confirmation of pythiosis. However, successful DNA extraction and PCR reaction rely on the amount of oomycetes present in the tissue [12, 19].

The most effective treatment is surgical resection of the mass with complete removal of the oomycote affecting the tissue or organ [13, 20–23]. In human cases of vascular pythiosis, early treatment and complete removal of the lesion with clear margins are probably the best indicator of survival [24]. Enlarged lymph nodes proximal to the lesion should be biopsied to determine if there is locoregional infection or widespread systemic infection. Antifungal therapy is not effective. The oomycote *P. insidiosum* has a cellular wall composed of cellulose and β-glucan and quasi-exempt of sterols such as ergosterols [1]. The latter are the main target of most antifungal agents including azoles (itraconazole, ketoconazole, fluconazole) and amphotericin B. Several sporadic responses have been reported in dogs [1, 25, 26]. There are no published guidelines on medical therapy against the oomycote *P. insidiosum* based on clinical trials. In this case report, no antifungal agent was administrated after surgical removal of the mass. A year after biopsy received on June 2016, a small scar tissue has been present at the surgical site with no size change. Considering the lack of regrowth for a year, the hyphal organisms were likely completely excised at the time of surgery. The cat is closely monitored.

## Conclusions

Only a few feline pythiosis cases have been reported and, when encountered, it caused diseases of the skin or gastrointestinal tract of Felidae. This case presents an unusual manifestation of feline pythiosis, representing the first involving the oral cavity. Pythiosis should be considered in the differential diagnosis of chronic infections and mass of the oral cavity in felines. Increasing awareness for this disease may lead to reduction of patient morbidity by early detection and intervention. Development of effective treatments is also needed to completely cure the disease.
